# Addressing diffusion behavior and impact in an epoxy–amine cure system using molecular dynamics simulations

**DOI:** 10.1038/s41598-022-26835-2

**Published:** 2023-01-04

**Authors:** Sung Hyun Kwon, Haisu Kang, Byeong-Joo Kim, Hyung Ik Lee, Jung Min Lee, Jungchul Kim, Seung Geol Lee

**Affiliations:** 1 School of Chemical Engineering, Pusan National University, Busan, 46241 Republic of Korea; 2 Research Institute of Industrial Technology, Pusan National University, Busan, 46241 Republic of Korea; 3 Agency for Defense Development, Yuseong P.O.Box 35, Daejeon, 34186 Republic of Korea; 4 Hankuk Carbon Co., Ltd., Miryang-si, Gyeongnam 50403 Republic of Korea; 5 Department of Organic Material Science and Engineering, Pusan National University, Busan, 46241 Republic of Korea

**Keywords:** Atomistic models, Composites

## Abstract

To deepen understanding of diffusion-controlled crosslinking, molecular dynamics (MD) simulations are carried out by taking the diffusion image of 3,3′-diamino diphenyl sulfone (3,3′-DDS) and polyethersulfone (PES) with epoxy resin varying temperatures from 393.15 to 473.15 K over crosslinking conversion of 0–85%. The diffusion of PES and 3,3′-DDS into the bulk increased with increasing the temperature as a result of enhanced mobility of the molecules when the difference between the glass-transition temperature (*T*_*g*_) and the curing temperature. Beyond the onset points of the converged crosslinking conversion ratio of 3,3′-DDS and PES, their diffusion properties are obviously restricted with crosslinking conversion ratio. At low crosslinking conversion ratios (> 10%), the diffusion coefficients of triglycidyl *p*-aminophenol (TGAP) were 1.1 times higher than those of diglycidyl ether of bisphenol F (DGEBF) because of the lower molecular weight of TGAP. On the other hand, the diffusion coefficients of TGAP decreased when the crosslinking ratio was up to ~ 60% because, compared with DGEBF, it had more functional groups available to react with the curing agent. At higher crosslinking ratios, the diffusion coefficients of both resins converged to zero as a result of their highly crosslinked structures.

## Introduction

Epoxy resins are versatile thermosetting materials, which have attracted attention worldwide because of their applications in diverse industries such as coatings, adhesives, industrial tools, aerospace, electronic materials, and biomedical systems^1–4^. In particular, epoxy resins have a high modulus, high specific strength, good heat resistance, and good electrical resistance^5–7^. So far, various kinds of epoxy resins with diverse functional groups as well as curing agents have been combined to satisfy industrial requirements. It has been recognized that the ultimate mechanical properties of epoxy systems are highly dependent on the state of cure or crosslink density, which takes root in the solution of epoxy resin and curing agents. On the other hand, highly crosslinked epoxy systems are prone to brittle failure when subjected to a strong external force. This is mainly arising from the fragile character of highly cured epoxy systems which behave as rigid yet brittle materials, exhibiting less resistance to crack initiation and growth. Therefore, various toughening agents with thermoplastic components such as polyethersulfone (PES)^8–10^, polysulfones^11,12^, polyimides^13^, and polyetherimides^14^ have been used in epoxy systems to improve the mechanical properties. General agreement was that the final properties of epoxy systems are determined by the dispersion of thermoplastic toughening agents within it. Therefore, it is important to understand the transport properties of thermoplastic toughening agents used in epoxy systems, not just the miscibility of epoxy systems. Moreover, the kinetic approach of an epoxy system is important not only for the final properties of an epoxy system but also for the performance of the composite system, which is semi-qualitatively measured by Cure Index^15,16^. Network formation and molecular structure of epoxy systems are determined by crosslinking reactions, which limit the diffusion of molecules^17^. Therefore, understanding the detailed kinetics of crosslinking is a key to manufacturing high-performance composites for special demands and applications, such as mechanically strong structures^18–23^.

Thermal analysis of curing has been systematically addressed by Jouyandeh and coworkers^24^. Nevertheless, a few have been known about the state of cure when gelation and vitrification phenomena are likely at high degrees of crosslinking conversion. As Jouyandeh et al. explained, the systems containing epoxy and curing agent undergo a chemically-controlled crosslinking at the early stage of the reaction where systems are almost liquid-like with low viscosity^25^. However, the thermal analysis fails to give useful information at later stages of the reaction where diffusion-controlled crosslinking is likely; even though that stage severely determines the ultimate properties of epoxy. In other words, understanding the diffusion properties when epoxy molecules are cured with curing agents is critical to achieving molecular-level control of the morphologies via the processing conditions. The molecular-level affinity of toughening agents with epoxy resins and curing agents also strongly influences the molecular diffusion behavior.

In a series of work, we conducted a full-atomistic molecular dynamics (MD) simulation to investigate the transport properties of the components of an epoxy resin system. Computational materials science techniques have been efficiently used to investigate the structural morphologies and diffusion properties of polymers in organic systems^26–32^. Other authors have recently investigated the mechanical properties of epoxy resin with curing agents^33–37^ and the phase separation of epoxy systems^38–41^ using computational materials science. Research into mechanical properties such as tensile and compressive stress–strain curves, Young’s modulus, shear modulus, glass-transition temperature (*T*_g_), and the phase separation of toughening agents with epoxy resin and curing agents is critical to achieving detailed atomic-level understanding of their behaviors. Moreover, investigating the detailed diffusion properties of molecules in an epoxy system when the epoxy resins are cured with curing agents is important to optimize the processing conditions.

Based on the above explanations, MD simulation can computationally image diffusion properties of components in the epoxy system and thermal properties as changing crosslinking conversion ratio and curing temperatures. Especially, the detailed diffusion properties of epoxy resin and curing agents with different sizes and molecular weights can calculate during the curing reaction^42^. Therefore, we constructed epoxy systems that contained two types of epoxy resin, a curing agent, and a toughening agent; then changed the temperature and conversion ratio on the basis of the MD simulation approach to elucidate their diffusion properties. The epoxy systems were composed of two resins to enable manipulation of their mechanical properties^43^ or their phase-separation behavior^44^. In particular, to elucidate the relationships between the diffusion coefficient and conversion ratio, we analyzed the diffusion properties of curing and toughening agents varying the temperature and the conversion ratio. In addition, we calculated the *T*_g_ of the epoxy system varying the conversion ratio to elucidate the relationship between the *T*_g_ and the conversion ratio and to predict the *T*_g_ as the conversion ratio was changed. Moreover, we calculated the change in the diffusion properties as a function of the number of epoxy groups available to promote the curing process of the epoxy system.

## Simulation methods and model preparation

### Computational details

**Figure 1:**
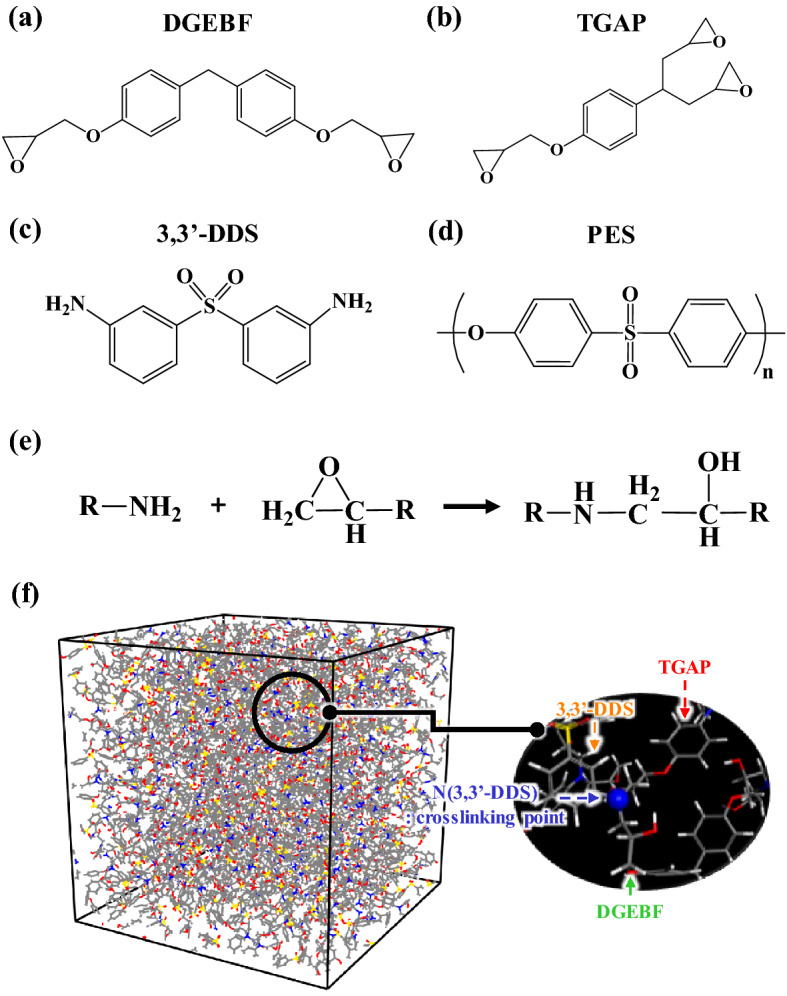
Molecular structures of (**a**) DGEBF, (**b**) TGAP, (**c**) 3,3′-DDS, and (**d**) PES. (**e**) Schematic of the crosslinking reaction between resins and amines. (**f**) The molecular structures of crosslinking structures with DGEBF, TGAP, and 3,3′-DDS.

Figure 1a–d show the chemical structures of diglycidyl ether of bisphenol F (DGEBF), triglycidyl *p*-aminophenol (TGAP), 3,3′-diamino diphenyl sulfone (3,3′-DDS), and polyether sulfone (PES) composing the epoxy systems, respectively. The Polymer Consistent Force-Field (PCFF)^45^ was used to describe the DGEBF, TGAP, 3,3′-DDS, and PES because the PCFF has been used to successfully describe a resin and curing agent in epoxy systems^33–36^. The total energies (E_total_s) of molecular structures of the epoxy systems were calculated using Eq. (1):

1 $$\begin{aligned} {E}_{{\mathrm{total}}}&={E}_{{\mathrm{vdW}}}+{E}_{{\mathrm{Q}}}+{E}_{{\mathrm{bond}}}+{E}_{{\mathrm{angle}}}+{E}_{{\mathrm{torsion}}}+{E}_{{\mathrm{inversion}}}+{E}_{{\mathrm{bond}}{-}{\mathrm{bond}}}+{E}_{{\mathrm{angle}}{-}{\mathrm{bond}}}\\ &\quad +{E}_{{\mathrm{angle}}{-}{\mathrm{angle}}}+{E}_{{\mathrm{angle}}{-}{\mathrm{torsion}}}+{E}_{{\mathrm{bond}}{-}{\mathrm{torsion}}} \end{aligned}$$

where *E*_vdW_, *E*_Q_, *E*_bond_, *E*_angle_, *E*_torsion_, *E*_inversion_, *E*_bond–bond_, *E*_angle–bond_, *E*_angle–angle_, *E*_angle–torsion_, and *E*_bond–torsion_ are the van der Waals, electrostatic, bond-stretching, angle-bending, torsion, out-of-plane bending angle, cross term between two bonds, coupling between bond and angle, two angles with a common bond, cross term for angle and torsion, and the bond and torsion angle, respectively. The MD calculations were performed using both the Large-scale Atomic/Molecular Massively Parallel Simulator (LAMMPS) code^46^ developed by S. Plimpton at Sandia National Laboratory and the Forcite modules in the Materials Studio software package from BIOBIA (San Diego, USA)^47^. The electrostatic interactions were calculated using the particle–particle particle–mesh method^48^. The velocity Verlet algorithm^49^ was used for MD simulations with time steps of 1.0 fs for integrating equations of atomic motions.

### Model preparation

The initial models of epoxy systems were constructed at a full-atomistic scale. The initial configuration of all components in the epoxy systems was established using the Monte Carlo method in Materials Studio^47^. Detailed molecular compositions used in our simulations of DGEBF, TGAP, 3,3′-DDS, and PES are summarized in Table 1. After the models were prepared, the initial models were optimized using an annealing procedure to obtain equilibrated structures efficiently and quickly^30,32,50^. The annealing procedure is described as follows: (a) The temperature of the initial structures of epoxy systems was gradually increased from 0 to 298.15 K over a period of 300 ps using NVT simulations. (b) The temperature was gradually increased from 298.15 to 600 K, accompanied by a gradual increase in the volume of the epoxy structures to 200% of their initial volume, over a period of 100 ps. (c) The temperature was maintained at 600 K over a period of 100 ps of the NVT simulations. (d) The temperature was then gradually decreased from 600 to 298.15 K, accompanied by a gradual decrease in the volume of the epoxy structures to their initial volume, over a period of 100 ps. (e) Steps (b)–(d) were repeated three times. (f) Epoxy systems were equilibrated over a period of 100 ps of NVT MD simulation at 298.15 K and 500 ps of isothermal–isobaric ensemble (NPT) simulation at 298.15 K and 1 atm. After completing the annealing procedure, we performed a crosslinking simulation to obtain cured epoxy structures with 0%, 10%, 30%, 50%, 70%, and 85% conversion at curing temperatures of 473.15, 453.15, 413.15, and 393.15 K on the basis of the schematic for the crosslinking reaction between resins and amines in Fig. 1e. After the crosslinking procedure, 10 ns of NPT MD simulation was performed to obtain the equilibrated structure in Fig. 1f, which is the crosslinked structures of DGEBF and TGAP with 3,3′-DDS. After the equilibrations, we analyzed the influence of the temperature and conversion ratio of the epoxy system using an additional 5 ns of NPT MD simulation for data collection. After data collection, the temperatures of the epoxy system were increased at 523.15 K over a period of 5 ns of NPT simulation to simulate the cooling simulation for initializing the *T*_g_ calculations. After the temperature was increased to 523.15 K, it was gradually decreased to 223.15 K over a period of 6 ns of NPT simulation to observe the change in density for the *T*_g_ calculation.

**Table 1:** Properties of the PES and epoxy system for simulation.

	DGEBF	TGAP	3,3′-DDS	PES (*M*_w_ = 1000)
Content ratio	50 wt%	50 wt%	30 phr	30 phr
Number of molecules	159	179	176	32

## Results and discussion

### Transport properties of the toughening agent and curing agent

**Figure 2:**
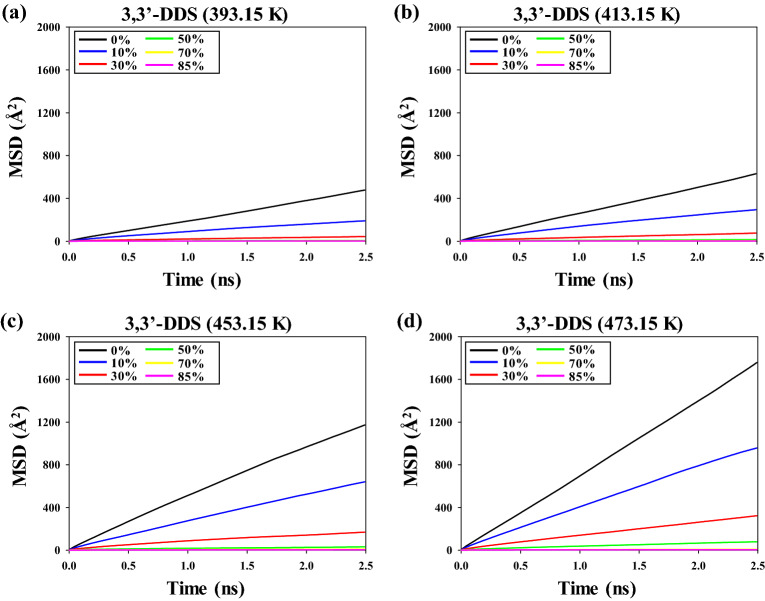
Mean squared displacement (MSD) of 3,3′-DDS at (**a**) 393.15 K, (**b**) 413.15 K, (**c**) 453.15 K, and (**d**) 473.15 K.

Figure 2 shows the mean squared displacement (MSD) of 3,3′-DDS at various temperatures and crosslinking conversion ratios. The slopes of the MSDs of 3,3′-DDS tend to decrease with increasing crosslinking conversion ratio, irrespective of temperature. In particular, the slopes of the MSDs of 3,3′-DDS decreased sharply as the crosslinking conversion ratio was increased from 0 to 50%. Therefore, the characteristics of the MSD of 3,3′-DDS were strongly affected by the crosslinking conversion ratio of the epoxy system. In addition, the MSD slope tends to increase with increasing temperature at the same crosslinking conversion ratio of the epoxy system because the components of the epoxy system exhibited increased molecular motion as the thermal energy of the system increased. However, the MSD slopes of 3,3′-DDS converged to zero when the crosslinking conversion ratio was greater than 50%, irrespective of temperature. The molecules in epoxy systems could not easily move to other sites at higher crosslinking conversion ratios, and the MSD of 3,3′-DDS was highly sensitive to structural changes of the epoxy systems because the monomers of the epoxy resin underwent a chemical reaction with the curing agent to form a network structure.

**Figure 3:**
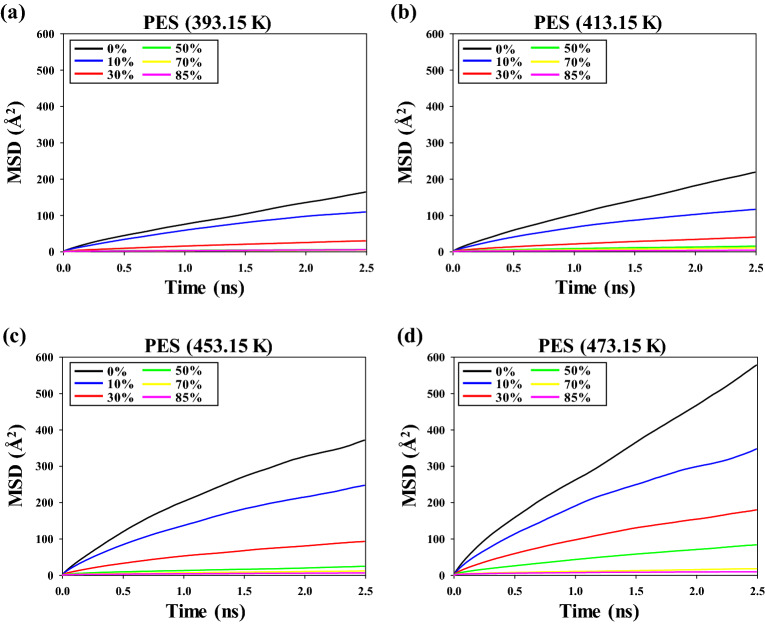
Mean squared displacement (MSD) of PES at (**a**) 393.15 K, (**b**) 413.15 K, (**c**) 453.15 K, and (**d**) 473.15 K.

Figure 3 shows the MSD of PES at various temperatures and crosslinking conversion ratios. With increasing crosslinking conversion ratio and temperature, the slopes of the MSDs of PES show a similar trend as the MSDs of 3,3′-DDS. In particular, the slopes of the MSD of PES decreased sharply as the crosslinking conversion ratio was increased from 0 to 50%. In addition, the MSD slopes of PES converged to zero when the crosslinking conversion ratio was greater than 50%, irrespective of temperature. PES molecules were added as a toughening agent because they do not chemically react with the epoxy resin or curing agents; however, the movement of PES molecules was restricted because of the rigidity of the thermosetting epoxy system.

The diffusion coefficients of 3,3′-DDS and PES in the epoxy system with various temperatures and crosslinking conversion ratios are based on Figs. 2 and 3, respectively. The diffusion coefficients can be obtained by Eq. (2):

2 $$D=\underset{t\to \infty }{{\mathrm{lim}}}\frac{1}{6t}\langle {(r(t)-r(0))}^{2}\rangle$$

where *r*(*t*) and *r*(0) indicate the positions of the target molecules at time *t* and at *t* = 0, respectively. The diffusion coefficients were greater at low crosslinking conversion ratios than at high crosslinking conversion ratios because the monomers and oligomers were major components of the epoxy system. However, the diffusion coefficients decreased with increasing crosslinking conversion ratio because the monomers of the epoxy resin reacted with curing agents to form network structures.

**Figure 4:**
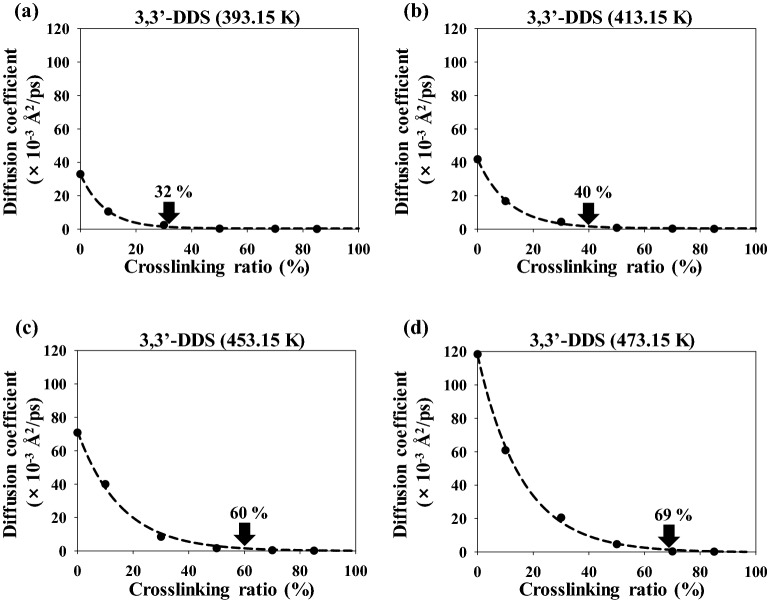
Diffusion coefficient of 3,3′-DDS at (**a**) 393.15 K, (**b**) 413.15 K, (**c**) 453.15 K, and (**d**) 473.15 K.

Figure 4 shows the diffusion coefficient of 3,3′-DDS as the temperature was increased from 393.15 to 453.15 K. The diffusion coefficient of 3,3′-DDS increased as the temperature increased at the same crosslinking conversion ratio because the molecular motions increased with increasing temperature. Irrespective of the temperature, the value of the diffusion coefficients substantially decreased with increasing crosslinking conversion ratio. The onset point of 3,3′-DDS, which is the crosslinking conversion ratio at which the diffusion coefficient becomes less than 0.1 × 10^−3^ Å^2^/ps per 1% of crosslinking conversion ratio, was 32%, 40%, 60%, and 69% at 393.15, 413.15, 453.15, and 473.15 K, respectively. We expect that higher temperatures of the epoxy system can expand the crosslinking conversion ratio range, thereby enabling control of the processing conditions of epoxy systems. Beyond the onset point of the converged crosslinking conversion ratio, the diffusion coefficients were close to zero and the molecules in the epoxy systems could not easily migrate to other sites. Thus, the liquid epoxy systems were transformed into an adhesive material with loss of fluidity and the distribution morphologies of the epoxy system were determined near the onset point of the diffusion coefficient.

**Figure 5:**
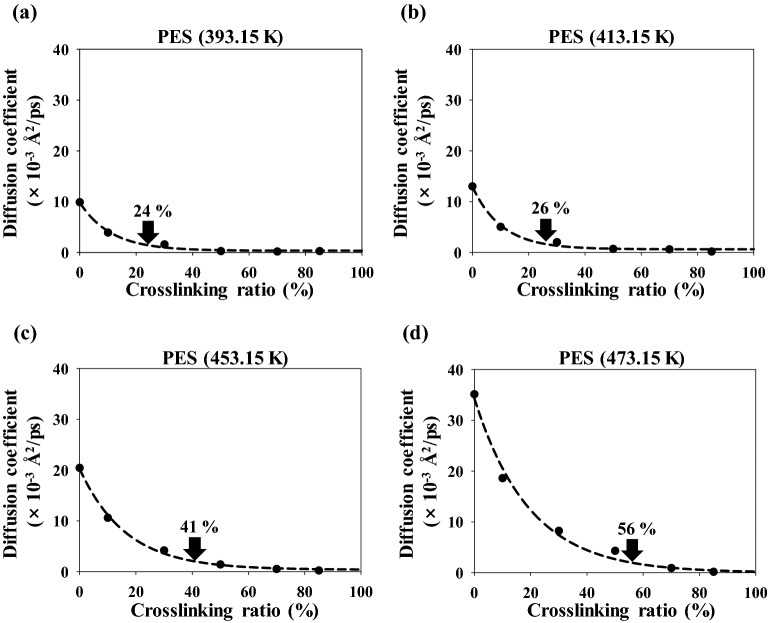
Diffusion coefficient of PES at (**a**) 393.15 K, (**b**) 413.15 K, (**c**) 453.15 K, and (**d**) 473.15 K.

Figure 5 shows the diffusion coefficient of PES as the temperature was increased from 393.15 to 473.15 K. The diffusion coefficients of PES show a trend similar to those of 3,3′-DDS with increasing crosslinking conversion ratio and temperature; specifically, the diffusion coefficients increase with increasing temperature at the same crosslinking conversion ratio. However, the diffusion coefficients decrease substantially with increasing crosslinking conversion ratio, irrespective of temperature. This tendency has similar to experimental data that the diffusion coefficients were significantly decreased at the initial crosslinking conversion ratio^17^. The onset points of PES were 24%, 26%, 41%, and 56% at 393.15, 413.15, 453.15, and 473.15 K, respectively. The onset points of PES have lower values than those of 3,3′-DDS at the same curing temperature because the PES molecules have a greater molecular weight than the 3,3′-DDS molecules. Moreover, the diffusion coefficient of 3,3’-DDS and PES were more affected by crosslinking conversion ratio at a lower temperature than at a higher temperature, and these results were well agreed with the experimental study^17^. In addition, understanding the relationship between the onset points of 3,3′-DDS and PES and the gelation point of the epoxy system is important for understanding the curing reaction and processing conditions of the epoxy system. The rate of the curing reaction decreased rapidly at low curing temperatures, likely because the diffusion properties of the molecules were limited before the crosslinking conversion ratio of the gelation points of our similar epoxy system reached approximately 50–60%^17,38,51,52^. By contrast, the curing reaction rate decreased more slowly at higher curing temperatures than at lower curing temperatures, likely because the values of the onset points of the diffusion coefficient of 3,3′-DDS are greater than the value of the gelation point. Therefore, the onset points of the diffusion of molecules should be compared with the difference between the *T*_g_ and the curing temperature according to the crosslinking conversion ratio because the difference between the *T*_g_ and the curing temperature strongly influences the diffusion of molecules.

### Glass-transition temperature with curing conversion

**Figure 6:**
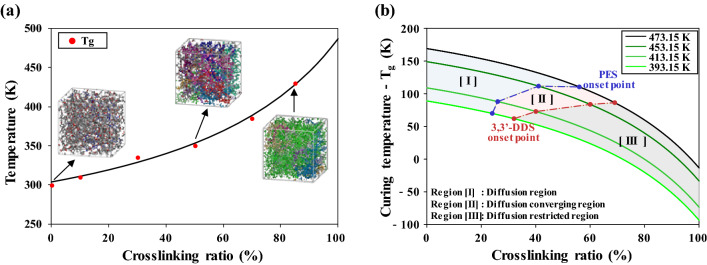
(**a**) Glass-transition temperature (*T*_g_) of the epoxy system as a function of the crosslinking ratio. The red dots represent the calculated *T*_g_ values, and the black line represents the fitting data. (**b**) Difference between the curing temperature and the *T*_g_, along with onset points of 3,3′-DDS and PES, for different curing temperature.

The diffusion properties of molecules not only affect the curing temperature but also strongly influence the *T*_g_. Figure 6a shows the *T*_g_ of the epoxy system at various conversion ratios. The relationship between the *T*_g_ and the conversion ratio, as proposed by Venditti and Gillham^53^, is represented by Eq. (3):^54^

3 $${\mathrm{ln}} ({T}_{{\mathrm{g}}})= \frac{(1-x){\mathrm{ln}} ({T}_{{\mathrm{g}}0})+\frac{\Delta {C}_{{\mathrm{P}}\infty }}{\Delta {C}_{{\mathrm{P}}0}}x{\mathrm{ln}} ({T}_{{\mathrm{g}}\infty })}{(1-x){\mathrm{ln}} ({T}_{{\mathrm{g}}0})+\frac{\Delta {C}_{{\mathrm{P}}\infty }}{\Delta {C}_{{\mathrm{P}}0}}x}$$

where *x*, *T*_g0_, and *T*_g∞_ represent the conversion ratio and the glass-transition temperatures of the fully unreacted and reacted epoxy system, respectively. Parameters Δ*C*_P0_ and Δ*C*_P∞_ represent the differences in heat capacity before and after the glass transitions of fully unreacted and reacted epoxy systems, respectively. The *T*_g_ of the epoxy system sharply increased with increasing crosslinking conversion ratio. During curing of the epoxy system, the curing temperatures should be higher than the *T*_g_ because the molecular mobility of amorphous polymers is increased at temperatures above their *T*_g_^55^. The difference between the curing temperature and the *T*_g_ is important because the diffusion properties of molecules were substantially increased as this difference increases^53^. Figure 6b shows the difference between curing temperature and the *T*_g_, along with the onset points of 3,3′-DDS and PES at different curing temperatures of 393.15 to 473.15 K. In region I, the diffusion coefficients of 3,3′-DDS and PES (Figs. 4 and 5, respectively) increase substantially with increasing curing temperature from 393.15 to 473.15 K because molecular motion increases with the increase in thermal energy and because the difference between the *T*_g_ and the curing temperature increases with decreasing crosslinking conversion ratio. In region II, the diffusion coefficients of 3,3′-DDS and PES converge to 0 with increasing crosslinking conversion ratio, which is the onset point. In region III, the diffusion of 3,3′-DDS and PES was restricted by an increase in the crosslinking conversion ratio above the onset point. The temperature difference between the curing temperature and the *T*_g_ also decreased with increasing crosslinking conversion ratio.

### Transport properties of DGEBF and TGAP

**Figure 7:**
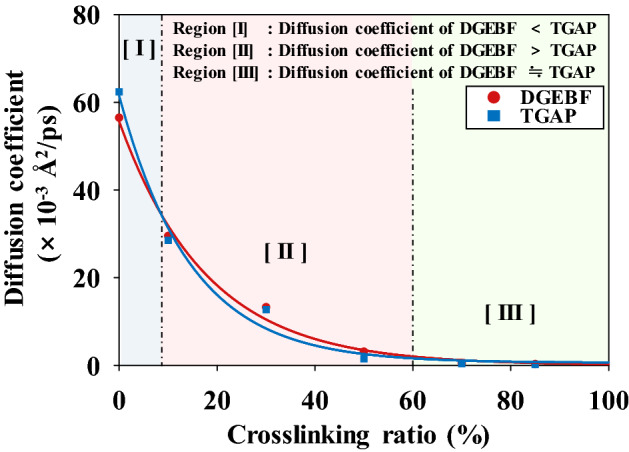
Diffusion coefficients of DGEBF and TGAP as functions of the crosslinking ratio at 180 °C. The red and blue dots represent the diffusion coefficients of DGEBF and TGAP, respectively. The red and blue lines represent the fitting data for DGEBF and TGAP, respectively.

The diffusion properties of the epoxy resins were measured according to the number of epoxy groups: two for DGEBF and three for TGAP. Figure 7 shows the diffusion coefficients of DGEBF and TGAP, along with their crosslinking conversion ratios at 453.15 K; the plot can be divided into three regions—I, II, and III—according to the correlation between the diffusion coefficients of DGEBF and TGAP. In region I, which includes the initial crosslinking conversion ratio of 0%, the diffusion coefficients of DGEBF are lower than those of TGAP because DGEBF has a higher molecular weight than TGAP. Therefore, the initial diffusion coefficients of the epoxy resins are affected by their molecular weight. In region II, the diffusion coefficients of DGEBF are higher than those of TGAP with increasing crosslinking conversion ratio of the epoxy system because the crosslinked molecular weight of DGEBF is lower than that of TGAP. Unlike TGAP, which offers a maximum of three crosslinking sites to curing agents, DGEBF can crosslink with two curing agents at most. Thus, TGAP has a higher probability of undergoing a curing reaction than DGEBF if both resins show a chemical reaction rate similar to amines and epoxy groups. Therefore, the crosslinked molecular weight with TGAP exhibits a higher probability of being heavier than that of DGEBF during the crosslinking reaction at a conversion ratio from ~ 10 to ~ 60%. From this perspective, the diffusion coefficients of TGAP decreased more than those of DGEBF with increasing crosslinking conversion ratio. These results show a similar trend as previously reported diffusion coefficients associated with amine and epoxy resins^42^. In region III, where the crosslinking conversion ratio is greater than 60%, the diffusion coefficients of DGEBF and TGAP converge to zero because not only DGEBF but also TGAP can react to form three-dimensional crosslinks with the amine group of 3,3′-DDS. The motion of the epoxy resins were greatly restricted by the rigidness of the thermosetting epoxy system after the crosslinking reactions with the epoxy resin and curing agent. These results indicate that the diffusion properties of a resin with different epoxy groups and various crosslinking conversion ratios are also a critical consideration in the design of the curing process of epoxy systems.

## Conclusion

We used full-atomistic MD simulations to investigate the diffusion kinetics of 3,3′-DDS and PES in an epoxy system as the temperature was varied from 393.15 to 413.15, 453.15, and 473.15 K and as the crosslinking conversion ratios were varied from 0 to 10%, 30%, 50%, 70%, and 85%. The MD simulations revealed that the diffusion properties of 3,3′-DDS and PES tend to increase with increasing temperature because of not only an increase in thermal energy but also an increase in the difference between their *T*_g_ values and the curing temperature. As the temperature was increased from 393.15 to 413.15, 453.15, and 473.15 K, the onset points of the crosslinking conversion ratio of 3,3′-DDS increased from 32 to 40%, 60%, and 69% and those of the crosslinking conversion ratio of PES increased from 24 to 26%, 41%, and 56%, respectively. At crosslinking conversion ratios greater than the onset points for 3,3′-DDS and PES, diffusion was substantially restricted not only because of high sensitivity to the structural changes of the epoxy systems but also because of a decrease in the difference between the *T*_g_ and curing temperature as a result of the increase in the *T*_g_ with increasing crosslinking conversion ratio. In addition, because of the smaller molecular weight of DGEBF compared with that of TGAP, the diffusion coefficients of TGAP were greater than those of DGEBF when the crosslinking conversion ratio was low (0–10%). However, the diffusion coefficients of TGAP decreased more than those of DGEBF as the crosslinking conversion ratio was increased to ~ 60% because of the greater number of functional groups for crosslinking reactions with curing agents, which can lead to an increase in the molecular weight. The diffusion coefficients of DGEBF and TGAP thereafter converged to zero when the crosslinking conversion ratio was greater than 60%. Our study indicated that the relationships between diffusion property and curing temperature of curing agents and toughening agents at various temperatures and crosslinking conversion ratios to be analyzed at the molecular level. Moreover, the diffusion properties of the epoxy resins were measured according to the number of epoxy groups and molecular weight. Therefore, we expect that MD simulations will aid in optimizing the curing conditions of epoxy systems at the molecular level.

## Data Availability

The datasets analyzed during the current study are not publicly available. However, they are available from the corresponding author on reasonable request.
