# Author Correction: Addressing diffusion behavior and impact in an epoxy–amine cure system using molecular dynamics simulations

**DOI:** 10.1038/s41598-023-28596-y

**Published:** 2023-01-25

**Authors:** Sung Hyun Kwon, Haisu Kang, Byeong‑Joo Kim, Hyung Ik Lee, Jung Min Lee, Jungchul Kim, Seung Geol Lee

**Affiliations:** 1 School of Chemical Engineering, Pusan National University, Busan, 46241 Republic of Korea; 2 Research Institute of Industrial Technology, Pusan National University, Busan, 46241 Republic of Korea; 3 Agency for Defense Development, Yuseong, P.O. Box 35, Daejeon, 34186 Republic of Korea; 4 Hankuk Carbon Co., Ltd., Miryang‑si, Gyeongnam 50403 Republic of Korea; 5 Department of Organic Material Science and Engineering, Pusan National University, Busan, 46241 Republic of Korea

Correction to: *Scientific Reports* 10.1038/s41598-022-26835-2, published online 04 January 2023

The Acknowledgment section in the original version of this Article was incorrect.

“This work was supported by the Agency For Defense Development Grant Funded by the Korean Government (UG200056GD). This study was financially supported by the 2022 Post-Doc. Development Program of Pusan National University”

now reads:

“This work was supported by the Agency For Defense Development Grant Funded by the Korean Government (UG200056GD).”

The original Article has been corrected.

